# Assessing the impact of non-pharmaceutical interventions (NPIs) and BCG vaccine cross-protection in the transmission dynamics of SARS-CoV-2 in eastern Africa

**DOI:** 10.1186/s13104-022-06171-4

**Published:** 2022-09-04

**Authors:** Chelsea Mbeke Kilonzo, Mark Wamalwa, Solange Youdom Whegang, Henri E. Z. Tonnang

**Affiliations:** 1 International Centre of Insect Physiology and Ecology (Icipe), P.O. Box 30772-00100, Nairobi, Kenya; 2 Department of Public Health, Faculty of Medicine and Pharmaceutical Sciences, University of Dschang, P.O Box: 96, Dschang, Cameroon; 3 Department of Biochemistry, Microbiology and Biotechnology, Kenyatta University, Nairobi, Kenya

**Keywords:** COVID-19, Time varying reproduction number, Bayesian hierarchical model, Epidemic trend

## Abstract

**Objective:**

The outbreak of the novel coronavirus disease 2019 (COVID-19) is still affecting African countries. The pandemic presents challenges on how to measure governmental, and community responses to the crisis. Beyond health risks, the socio-economic implications of the pandemic motivated us to examine the transmission dynamics of COVID-19 and the impact of non-pharmaceutical interventions (NPIs). The main objective of this study was to assess the impact of BCG vaccination and NPIs enforced on COVID-19 case-death-recovery counts weighted by age-structured population in Ethiopia, Kenya, and Rwanda. We applied a semi-mechanistic Bayesian hierarchical model (BHM) combined with Markov Chain Monte Carlo (MCMC) simulation to the age-structured pandemic data obtained from the target countries.

**Results:**

The estimated mean effective reproductive number (R_t_) for COVID-19 was 2.50 (C1: 1.99–5.95), 3.51 (CI: 2.28–7.28) and 3.53 (CI: 2.97–5.60) in Ethiopia, Kenya and Rwanda respectively. Our results indicate that NPIs such as lockdowns, and curfews had a large effect on reducing R_t_. Current interventions have been effective in reducing R_t_ and thereby achieve control of the epidemic. Beyond age-structure and NPIs, we found no significant association between COVID-19 and BCG vaccine-induced protection. Continued interventions should be strengthened to control transmission of SARS-CoV-2.

**Supplementary Information:**

The online version contains supplementary material available at 10.1186/s13104-022-06171-4.

## Introduction

The emergence of COVID-19 pandemic was expected to have devastating consequences in Africa due to the weak healthcare systems [1–3]. However, fatalities have remained low and most cases are asymptomatic [4]. This is attributed to previous exposure to epidemics such as Ebola, demographic factors, host genetics and environmental factors [2, 5]. Apart from Africa’s young population, Bacillus Calmette − Guérin (BCG) vaccine against tuberculosis was proposed to reduce the severity of COVID-19 [6–8].

Most countries implemented NPIs to limit human-to-human transmission of SARS-CoV-2 and therefore lower the reproduction number (R_0_)—the number of secondary infections acquired from a primary case [9–11]. It was imperative to quantify enforced NPIs in terms of their efficacy and appropriate use to influence and improve public health policy. Indeed, several models have been used to unravel COVID-19 [11–13].

The aim of this study was to examine the association between age-structure and BCG vaccine-induced protection from COVID-19 and to assess the impact of NPIs implemented in Ethiopia, Kenya, and Rwanda.

## Main text

### Methods

#### Data sources

COVID-19 data (2020–2021) were obtained from the World Health Organization (WHO) and the Johns Hopkins University (JHU) repositories [14, 15]. Population data were sourced from United Nations (UN) records [16] while BCG vaccination records from 1980–2019 (both sexes combined) were obtained from the WHO [17, 18].

BCG vaccination data were segmented into 10-year age-groups, and the mean percentage vaccination coverage (*pvc*) was calculated, assigning zero coverage to age-groups above 40 years [19], (Additional file 1: Table S1). *pvc* was used to infer the number of BCG-vaccinated individuals (*N*_*m*_) in country *m* (Eq. 11).

COVID-19 data were split into two age-groups, 0–39 and 40 and above. This was based on the fact that BCG vaccination was introduced in EACs in the early 1980s, and therefore, only individuals aged below 39 years were assumed to be vaccinated by 2019 [19]. Finally, implementation dates of NPIs were obtained from the respective government websites and media houses (Additional file 1: Table S2).

#### Model formulation

At the onset of the pandemic, the Imperial College London (ICL) proposed a BHM that uses observed deaths to infer the true number of infections [11]. Deaths were expressed as a function of infection-fatality-ratio, infection-to-onset, and onset-to-death distributions [11]. Infections were expressed as a product of the time-varying reproduction number (R_t_) with a discrete convolution of previous infections weighted by an infection-to-onset distribution specific to SARS-CoV-2 [11]. R_t_ was inferred from the initial R_0_ before interventions and the effect sizes from the interventions [11]. The ICL model has been applied in several studies [11, 20–22] and its’ adapted structure used in this study is shown in Additional file 1: Figure S1.

#### Infection model specification

The infected population (*c*) on day (*t*) was modelled as a discrete renewal process. The model was initialized by a serial interval distribution (*g*) with density *g*(τ), whereby *g* is gamma distributed with a mean of 6.5 and standard deviation of 0.62 (Eq. 1) [23]. *g* is shared across all the countries [11].

1 $$g\sim Gamma\,(6.5,0.62)$$

The number of infections (*c*_*t*,*m*_) on day *t*, in country, *m*, were approximated by a discrete convolution function (Eq. 2).

2 $$c_{{t,m}} = R_{{t,m}} \sum\limits_{{t = 0}}^{{t - 1}} {c_{{\tau ,m}} g_{{t - \tau }} }$$

Daily g was discretized by the serial interval (${g}_{s}$) (Eq. 3).

3 $$g_{s} = {\text{ }}\int\limits_{{\tau = s - 0.5}}^{{s + 0.5}} {g(\tau )d(\tau )} {\text{ for s}} = {\text{2}},{\text{3 and }}g_{1} = {\text{ }}\int\limits_{{\tau = 0}}^{{1.5}} {g(\tau )d(\tau )}$$

The current number of infections were determined by infections in the previous days, weighted by ${g}_{s}$.

${R}_{t,m}$ is a function of interventions (*I*_*k*,*t*,*m*_) imposed at time *t* in country *m* (Eq. 4) [11].

4 $$R_{{t,m}} = R_{{0,m}} {\text{ exp}}( - \sum _{{k = 1}}^{7} \alpha _{k} I_{{k,t,m}} )$$

The implemented intervention (*I*) is denoted by k which is 1 if k is enforced in country *m* at time *t*, and 0 otherwise. Exponentiation of Eq. 4 constrains *R*_0, *m*_ to be positive. Further, α_1, …7_ determines the impact of each intervention on *R*_*t*, *m*_. Prior distributions of α are Gamma distributed, ${\alpha }_{k}\sim Gamma(0. \mathrm{5,1})$. R_0_ assumes a prior distribution specified below (Eq. 5).

5 $$R_{0} \sim Normal(2.4,|k|)\,{\text{where}}\,k\sim Normal\,({\text{0}},{\text{0}}.{\text{5}})$$

#### Death model specification

Daily deaths (d_t, m_) for days *t ∈ *{1, …, n} and countries *m ∈ {1, …, p}* were projected using a function d_t, m_ = E[d_t, m_] whereby d_t, m_ represents daily deaths, and it follows a negative binomial distribution with mean = d_t, m_ and variance = ${d}_{t,m}+ {d}_{t,m}^{2}/{\Psi }_{1}$, where ψ_1_ follows a positive half-normal distribution (Eq. 6a and 6b) [11].

6a $$\begin{gathered} d_{{t,m}} \sim Negative\,Binomial\,(d_{{t,m}} ,d_{{t,m}} + \frac{{d_{{t,m}} ^{2} }}{\Psi }) \hfill \\ \hfill \\ \end{gathered}$$

6b $$\Psi \sim {Normal}^{+}(\mathrm{0,5})$$

Observed deaths are associated with cases using the infection-fatality-ratio (*ifr*, probability of death given infection) of 0.1% and the infection-to-death (*π*) distribution [20]. The model applies an adjusted *ifr* (*ifr*_*a*_) that incorporates the attack rate and the population size [20]. Therefore, ${ifr}_{\alpha }=\frac{{AR}_{0-39}}{{AR}_{\alpha }}$ where ${AR}_{0-39}$ is the age-group-specific attack rate. ${AR}_{\alpha }=\frac{{c}_{\alpha }}{{N}_{\alpha }}$ where, ${c}_{\alpha }$ is the number of infections in age-group α, and ${N}_{\alpha }$ the population size. The infection-to-death (π) distribution consists of infection-to-onset (π′) and onset-to-death distributions. π was initialized using values from Verity et al.[11, 20]. Infection-to-onset is Gamma distributed with a mean of 5.1 days and coefficient of variation of 0.86 while onset-to-death is also Gamma distributed with a mean of 18.8 days and a coefficient of variation of 0.45 (Eq. 7﻿) [11].

7 $${\pi }_{m}\sim {ifr}_{m}.(Gamma\left(5.\mathrm{1,0.86}\right)+Gamma(18.8, 0.45))$$

The expected number of deaths d_t, m_, on day *t*, in country *m* was estimated by Eq. 8.

8 $$d_{{t,m}} = \sum\limits_{{t = 0}}^{{t - 1}} {c_{{\tau ,m}} \pi _{{t - \tau }} }$$

where, ${c}_{\tau ,m}$ is the number of new infections on day τ in country *m.* π_*m*_ is discretized via Eq. 9

9 $$\pi _{{s,m}} = \int\limits_{{\tau = s - 0.5}}^{{s + 0.5}} {\pi _{m} (\tau )d(\tau )\,} {\text{for}}\,{\text{s}} = {\text{2}},{\text{3}}\,{\text{and}}\,\pi _{{1,m}} = \int\limits_{{\tau = 0}}^{{1.5}} {\pi _{m} (\tau )d}$$

#### BCG vaccine-induced protection

To assess vaccine-induced protection from COVID-19, the number of BCG-vaccinated individuals (*N*_*m*_) in country *m* was assumed to have anti-SARS-CoV-2 antibodies. *N*_*m*_ was applied as a scaling factor to estimate susceptible individuals (*S*_*t, m*_) on day *t*, in country *m* (Eq﻿. 10).

10 $${S}_{t,m}=1- \frac{\sum_{j=0}^{t-1}{i}_{j,m}}{{N}_{m}}$$

The number of infections (i_*t*, *m*_) on day *t*, in country *m*, was estimated by a discrete convolution function (Eq. 11).

11 $$i_{{t,m}} = S_{{t,m}} R_{{t,m}} \sum\limits_{{t = 0}}^{{t - 1}} {c_{{\tau ,m}} g_{{t - \tau }} }$$

The daily g was discretized by ${g}_{s}$ distribution (Eq. 3). Similarly, we computed ${R}_{t,m}$ weighted by the interventions (*I*_*k*, *t*,*m*_) at time *t* in country *m* (Eq. 4). The expected number of deaths (d_t, m_) on day *t*, for country *m* was estimated by Eq. 12.

12 $$d_{{t,m}} = \sum\limits_{{t = 0}}^{{t - 1}} {i_{{\tau ,m}} \pi _{{t - \tau }} }$$

where, ${i}_{\tau ,m}$ is the number of new infections on day τ in country *m.* π_t-τ_ is discretized via Eq. 9.

#### The SEIR and eSIR compartmental models

We explored an extension of the susceptible-exposed-infectious-recovered (SEIR) model to compute R_t_ [24]. Ordinary differential equations (ODE) that describe the dynamics of this model, are extensively described in [24]. Moreover, we used the extended SIR (eSIR) model to predict R_t_ values [25, 26].

#### Model implementation

Modelling was implemented in R (version 4.0.4) using ICL covid19model version 10 [11]. The ICL model was run in Stan R package using 500 iterations [11, 27]. Computation of the Root Mean Square Error (RMSE) and Mean Absolute Error (MAE) was executed in the *ehaGoF* R package [28] while the SEIR model was executed using the SEIR-fansy R package [24].

#### Model validation and comparison

The reliability of the ICL model was assessed by comparing model predictions against the observed data between 06/16/20 and 04/11/2021 using RMSE and MAE metrics [29, 30]. Additional validation was performed using an approach suggested by Flaxman et al. through an importance sampling leave-one-out cross validation scheme [11, 21, 28]. Moreover, we compared the ICL model with compartmental models using the predicted R_t_ and case-death-recovery counts [24–26].

### Results

#### Scenario analysis of COVID-19 trends

We evaluated the effectiveness of NPIs under two scenarios: presence or absence of an age-structured population and BCG vaccination. Assuming that the population is homogenous and not structured by age, the ICL model estimated R_t_ values were 2.50 (C1: 1.99–5.95), 3.51 (CI: 2.28–7.28) and 3.53 (CI: 2.97–5.60) in Ethiopia, Kenya and Rwanda respectively (Table 1).

**Table 1:** Comparison of predicted case-death counts and time-varying reproduction number (R_t_)

Country	Cases	Deaths	ICL (R_t_)^1^	eSIR (R_t_)^2^	SEIR (R_t_)^3^	RMSE^4^	MAE^5^
**Ethiopia (ET)**	**363,714**	**6412**	**2.50 (1.9–5.95)**	**2.75**	**2.98**	**4.257**	**0.027**
ET + BCG	363,714	6412	1.67 (1.5**–**3.19)			4.138	0.026
ET(40 +) + BCG	134,716	5408	5.25 (3.3**–**8.16)			3.558	0.026
**Kenya (KE)**	**252,938**	**5266**	**3.51 (2.8–7.28)**	**2.70**	**2.51**	**4.652**	**0.033**
KE + BCG	252,938	5266	5.34 (3.5**–**7.99)			31.014	0.219
KE(0–39) + BCG	159,259	821	5.18 (3.8**–**7.87)			33.642	1.449
KE(40 +) + BCG	93,679	4445	5.15 (3.2**–**7.67)			3.701	0.031
**Rwanda (RW)**	**99,559**	**1322**	**3.53 (2.7–5.60)**	**3.10**	**2.03**	**0.932**	**0.051**
RW + BCG	99,559	1322	6.32 (4.53**–**13.34)			0.962	0.053
RW(0–39) + BCG	62,693	180	5.21 (3.5**–**8.62)			0.151	0.057
RW(40 +) + BCG	36,866	1142	5.93 (4.4**–**9.97)			0.834	0.052

^ 1^ICL (Rt) - Imperial College London (ICL) model estimates of the time-varying reproduction number (R_t_). ^2^eSIR (R_t_) - the extended susceptible-infected-removed (eSIR) compartmental model estimates of the time-varying reproduction number. ^3^SEIR(Rt) - susceptible-exposed-infectious-recovered (SEIR) compartmental model estimates of the time-varying reproduction number. ^4^RMSE measures the model (ICL) prediction accuracy against the observed data in a regression analysis. It is the Root of the Mean of the Square of Errors between the predicted and the observed COVID-19 cases and deaths. ^5^MAE measures the accuracy of the model fit in terms of performance in its predictions - the Mean of Absolute value of Errors between the predicted and the observed COVID-19 cases and deaths. The mean R_t_ values projected by the ICL model overlapped with the SEIR and eSIR models. However, the ICL model tends to overestimate R_t_ values while the SEIR model had less variability (Table 1) [31]

We observed a good model fit between the predicted and the reported cases across East Africa. Larger RMSE values, particularly in Kenya, indicate a wider divergence between the predicted and observed values. Similarly, the computed MAE values ranged between 0.026–1.449 (Table 1). In general, lower RMSE and MAE values provide better support for the model fit.

**Fig. 1:**
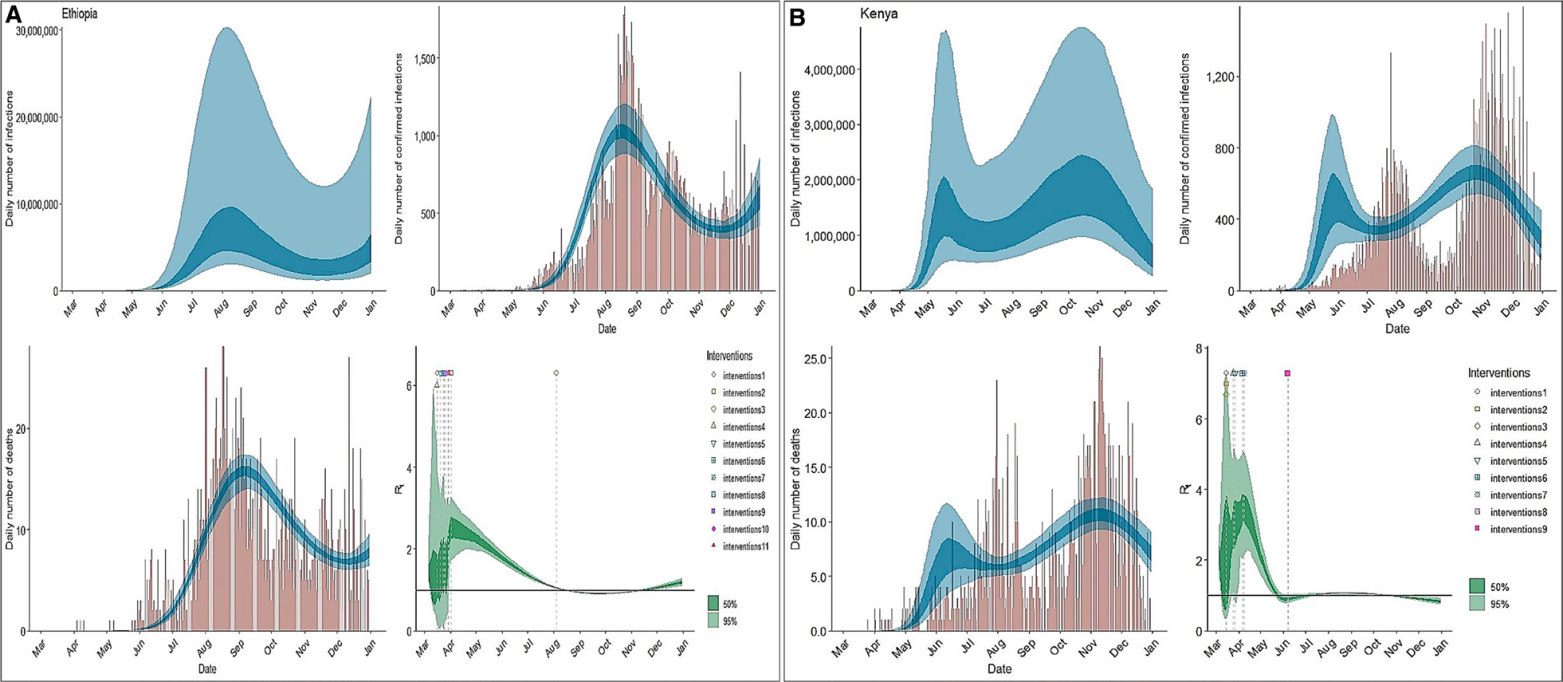
Country-level estimates of infections, deaths and R_t_. *Top*: daily number of infections, brown bars are reported infections, blue bands are predicted infections, dark blue 50% credible interval (CI), light blue 95% CI. *Bottom-left*: daily number of deaths, brown bars are reported deaths, blue bands are predicted deaths. *Bottom-right*: time-varying reproduction number (R_***t***_), dark-green 50% CI, light-green 95% CI

**Fig. 2:**
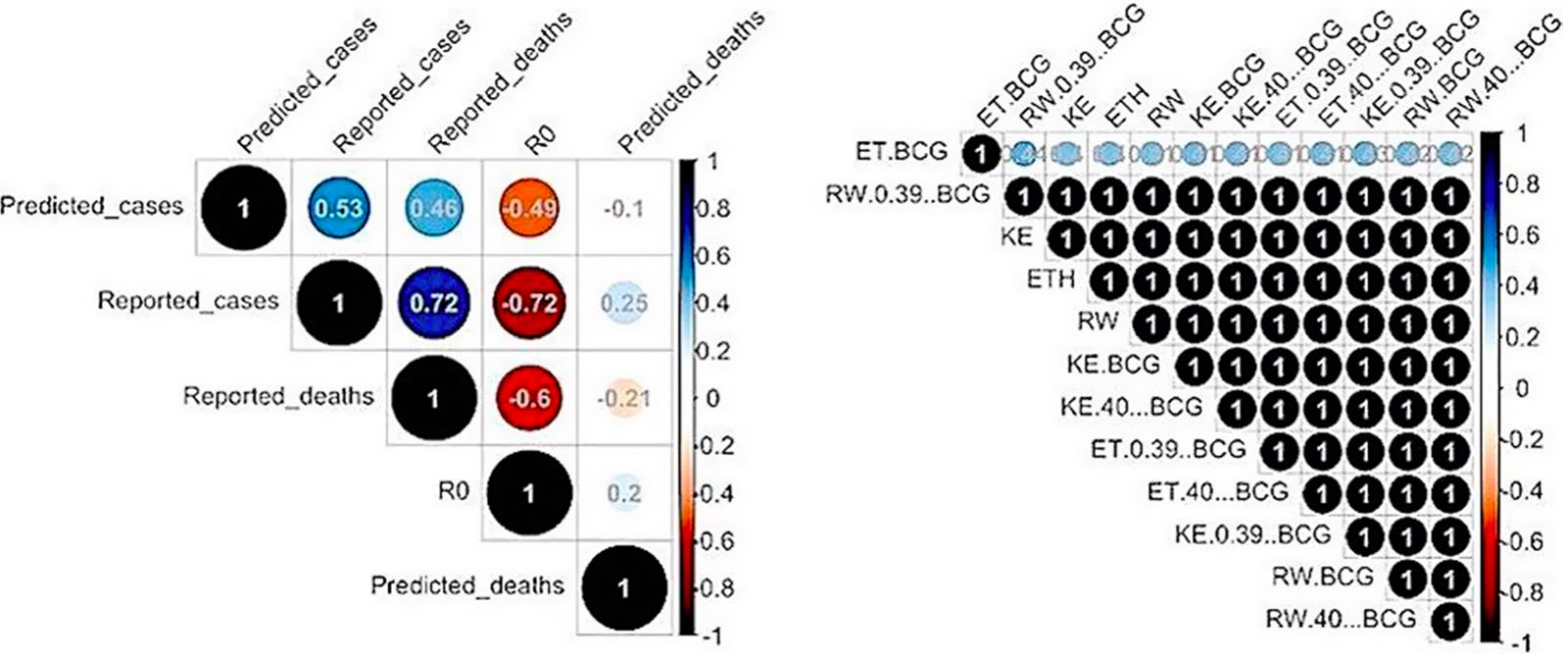
The association between BCG vaccination, R_0_ and the case-death counts. **A** Association between R_0_ and case-death counts; **B** Association between age-structure, case-death counts and R_0_

Additionally, our results indicate that lockdowns and curfews profoundly reduced the R_t_ in Kenya (Fig. 1A) while in Ethiopia, the declaration of emergency and regional lockdowns reduced human-to-human transmissions (Fig. 1B). The dusk-to-dawn curfews in Rwanda had the most effect in lowering R_t_. Beyond age-structure, we found no significant association between COVID-19 and BCG-vaccine induced protection (Fig. 2 and Additional file 1: Figure S2, S3).

## Discussion

Parameters such as R_t_ and serial interval are estimators of the disease extent in a given country and they inform policy-makers about the most effective interventions [32]. Our findings show that, Ethiopia, Kenya and Rwanda were at a critical point in 2020, whereby R_t_ values remained above 2, however, while infections were high, fatalities remained low. Indeed, this is an African paradox where COVID-19 infections have consistently remained high while fatalities are low [7].

Beyond under-estimation of the disease extent, multiple factors have been associated with the low fatalities, namely, herd immunity due to anti-SARS-CoV-2 antibodies, climate, comorbidities and demographic structure [5]. These factors have not been studied conclusively to establish their association with COVID-19 [5].

While BCG vaccine offers cross-protection against other diseases, it has also been proposed to reduce the severity of COVID-19 [6, 33]. However, our findings show that there is no linkage between BCG vaccination and COVID-19 prevalence. In fact, the WHO did not find evidence of BCG vaccine-induced protection from COVID-19 [34].

Africa’s predominantly young population, with fewer comorbidities has been associated with the low prevalence of COVID-19 relative to other continents [35, 36]. Indeed, we observed a negative correlation between R_t_ and age-structured case-death counts. It is noteworthy that, in this study, the susceptible population was segmented according to age and BCG vaccination status prior to estimation of posterior parameters. Consequently, age had a confounding effect on R_t_ and case-death counts wherein the effect of BCG vaccination could not be separated from the effect of age-structure. Generally, majority of COVID-19 cases were identified to be in the age group 30–39 while most deaths comprised of those aged above 40 [14].

## Limitations

The ICL model uses observed deaths to infer the true number of infections [11]. While this approach overcomes uncertainties associated with asymptomatic cases and low testing in the African context, inferring infections from deaths to estimate the burden of the disease is challenging given the low mortality recorded in Africa. Further, some parameters were set by assumption or used values from literature, which significantly affect the parameter estimation.

## Supplementary Information

**Additional file 1: Table S1.** Country-level percentage coverage of Bacillus Calmette–Guérin (BCG) vaccination in 2019, by age group. **Table S2.** Country-level dates of implementation of non-pharmaceutical interventions. **Figure S1.** Schematic overview of the Imperial College London (ICL) model adopted in this study [11]. **Figure S2.** Country-level estimates of infections, deaths and R_t_ in Ethiopia. *Scenarios*: A) The population is not BCG vaccinates, homogenous and not structured by age; B) BCG vaccinated population aged 39 years and below; C) BCG vaccinated population aged 40 years and above. *Top*: daily number of infections, brown bars are reported infections, blue bands are predicted infections, dark blue 50% credible interval (CI), light blue 95% CI. *Bottom-left*: daily number of deaths, brown bars are reported deaths, blue bands are predicted deaths. *Bottom-right*: time-varying reproduction number (R_***t***_), dark-green 50% CI, light-green 95% CI. **Figure S3.** Country-level estimates of infections, deaths and R_t_ in Kenya. *Scenarios*: A) The population is not BCG vaccinates, homogenous and not structured by age; B) BCG vaccinated population aged 39 years and below; C) BCG vaccinated population aged 40 years and above. *Top*: daily number of infections, brown bars are reported infections, blue bands are predicted infections, dark blue 50% credible interval (CI), light blue 95% CI. *Bottom-left*: daily number of deaths, brown bars are reported deaths, blue bands are predicted deaths. *Bottom-right*: time-varying reproduction number (R_***t***_), dark-green 50% CI, light-green 95% CI.

## Data Availability

The data of cumulative number of COVID-19 infected cases are available from COVID-19 Data Repository by the Johns Hopkins University Center for Systems Science and Engineering (JHU CCSE) at https://github.com/CSSEGISandData/COVID-19. The R packages used in this study are publicly available at https://github.com/ImperialCollegeLondon/covid19model, https://github.com/lilywang1988/eSIR and at https://github.com/umich-biostatistics/SEIRfansy.

## Abbreviations

COVID-19: Coronavirus disease 2019
BCG: Bacillus Calmette-Guérin Vaccine
SARS-CoV-2: Severe acute respiratory syndrome coronavirus 2
HIV: Human immunodeficiency virus
NPIs: Non-pharmaceutical intervention(s)
SIR: Susceptible, infected, recovered
eSIR: Extended susceptible, infected, removed
SEIR: Susceptible, exposed, infected, recovered
R_0_: The basic reproduction number
R_t_: Time-varying reproduction number
MCMC: Markov chain Monte Carlo
RMSE: Root Mean square error
MAE: Mean absolute error
JHU: Johns Hopkins University
ICL: Imperial College London
EACs: East African Countries
CI: Credible interval
