# Supplementary material for: Assessing the impact of non-pharmaceutical interventions (NPIs) and BCG vaccine cross-protection in the transmission dynamics of SARS-CoV-2 in eastern Africa
Source: BMC Res Notes. 2022 Sep 4;15:283. doi: 10.1186/s13104-022-06171-4 (PMC9440862; doi:10.1186/s13104-022-06171-4)
Supplement: Supplementary file 1 — Additional file 1: Table S1. Country-level percentage coverage of Bacillus Calmette–Guérin (BCG) vaccination in 2019, by age group. Table S2. Country-level dates of implementation of non-pharmaceutical interventions. Figure S1. Schematic overview of the Imperial College London (ICL) model adopted in this study [11]. Figure S2. Country-level estimates of infections, deaths and Rt in Ethiopia. Scenarios: A) The population is not BCG vaccinates, homogenous and not structured by age; B) BCG vaccinated population aged 39 years and below; C) BCG vaccinated population aged 40 years and above. Top: daily number of infections, brown bars are reported infections, blue bands are predicted infections, dark blue 50% credible interval (CI), light blue 95% CI. Bottom-left: daily number of deaths, brown bars are reported deaths, blue bands are predicted deaths. Bottom-right: time-varying reproduction number (Rt), dark-green 50% CI, light-green 95% CI. Figure S3. Country-level estimates of infections, deaths and Rt in Kenya. Scenarios: A) The population is not BCG vaccinates, homogenous and not structured by age; B) BCG vaccinated population aged 39 years and below; C) BCG vaccinated population aged 40 years and above. Top: daily number of infections, brown bars are reported infections, blue bands are predicted infections, dark blue 50% credible interval (CI), light blue 95% CI. Bottom-left: daily number of deaths, brown bars are reported deaths, blue bands are predicted deaths. Bottom-right: time-varying reproduction number (Rt), dark-green 50% CI, light-green 95% CI. [file 13104_2022_6171_MOESM1_ESM.docx]

**Additional file: Supporting Figures and Tables**

**Assessing the impact of non-pharmaceutical interventions (NPIs) and BCG vaccine cross-protection in the transmission dynamics of SARS-CoV-2 in eastern Africa**

Chelsea Mbeke Kilonzo^1^, Mark Wamalwa^1,3*^, Solange Youdom Whegang^2^, Henri E.Z. Tonnang^1^

^1^International Centre of Insect Physiology and Ecology (*icipe*), P.O. Box 30772-00100, Nairobi, Kenya

^2^Department of Public Health, Faculty of Medicine and Pharmaceutical Sciences, University of Dschang, P.O Box: 96, Dschang, Cameroon

^3^Department of Biochemistry, Microbiology and Biotechnology, Kenyatta University, Nairobi, Kenya

*Corresponding author email address: [mwamalwa@icipe.org](mailto:mwamalwa@icipe.org)

**Table S1**: Country-level percentage coverage of Bacillus Calmette–Guérin (BCG) vaccination in 2019, by age group.

| **Country** | **0-9** | **10-19** | **20-29** | **30-39** | **40-49** | **50-59** | **60+** | **Mean BCG Vaccination (%)** |
| --- | --- | --- | --- | --- | --- | --- | --- | --- |
| Ethiopia | 72.2 | 63.4 | 50 | 14.9 | 0 | 0 | 0 | 28.64 |
| Kenya | 94.6 | 91.6 | 95.1 | 50.4 | 0 | 0 | 0 | 47.39 |
| Rwanda | 98.6 | 88 | 86.1 | 66.6 | 0 | 0 | 0 | 48.47 |

**Table S2**: Country-level dates of implementation of non-pharmaceutical interventions.

| **Country** | **Non-Pharmaceutical Intervention** | **Date of enforcement** |
| --- | --- | --- |
| **Ethiopia** | School closure primary | 3/16/2020 |
|  | School closure university | 3/24/2020 |
|  | Public events | 3/16/2020 |
|  | Work home | 3/25/2020 |
|  | Travel restriction 1 | 3/20/2020 |
|  | Travel restriction 2 | 3/29/2020 |
|  | Prisoners released | 3/26/2020 |
|  | Prisoners released | 4/2/2020 |
|  | Lockdown borders | 3/23/2020 |
|  | Lockdown regional | 3/30/2020 |
|  | Emergency | 8/4/2020 |
| **Kenya** | School closure | 3/15/2020 |
|  | Public events | 3/15/2020 |
|  | Travel restrictions | 3/15/2020 |
|  | Travel restrictions international | 3/25/2020 |
|  | Curfew 7pm-5am | 3/27/2020 |
|  | Curfew 9pm-4am | 6/6/2020 |
|  | Curfew 8pm-4am regional | 3/26/2021 |
|  | Lockdown regional | 4/6/2020 |
|  | Lockdown regional | 4/8/2020 |
|  | Vaccination | 3/5/2021 |
| **Rwanda** | School closure 1 | 3/21/2020 |
|  | School closure 2 | 1/18/2021 |
|  | Public events | 3/21/2020 |
|  | Travel restriction | 3/20/2020 |
|  | Travel restriction | 1/18/2020 |
|  | Curfew 6am 4pm | 3/18/2020 |
|  | Vaccination | 3/5/2021 |


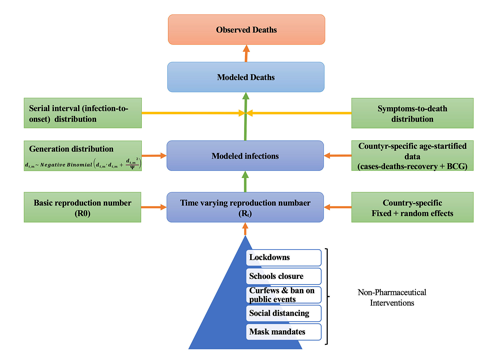


**Figure S1**. Schematic overview of the Imperial College London (ICL) model adopted in this study [11].


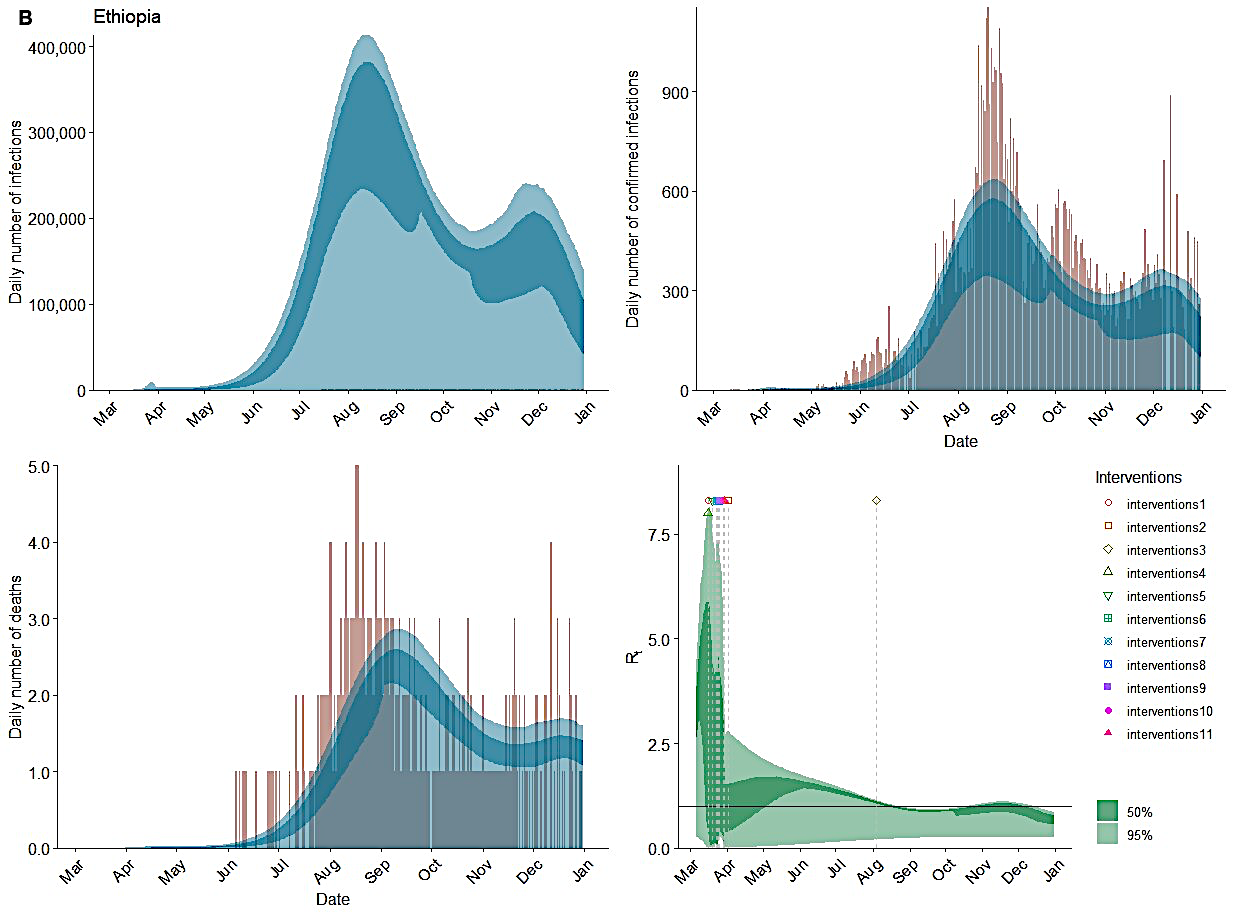


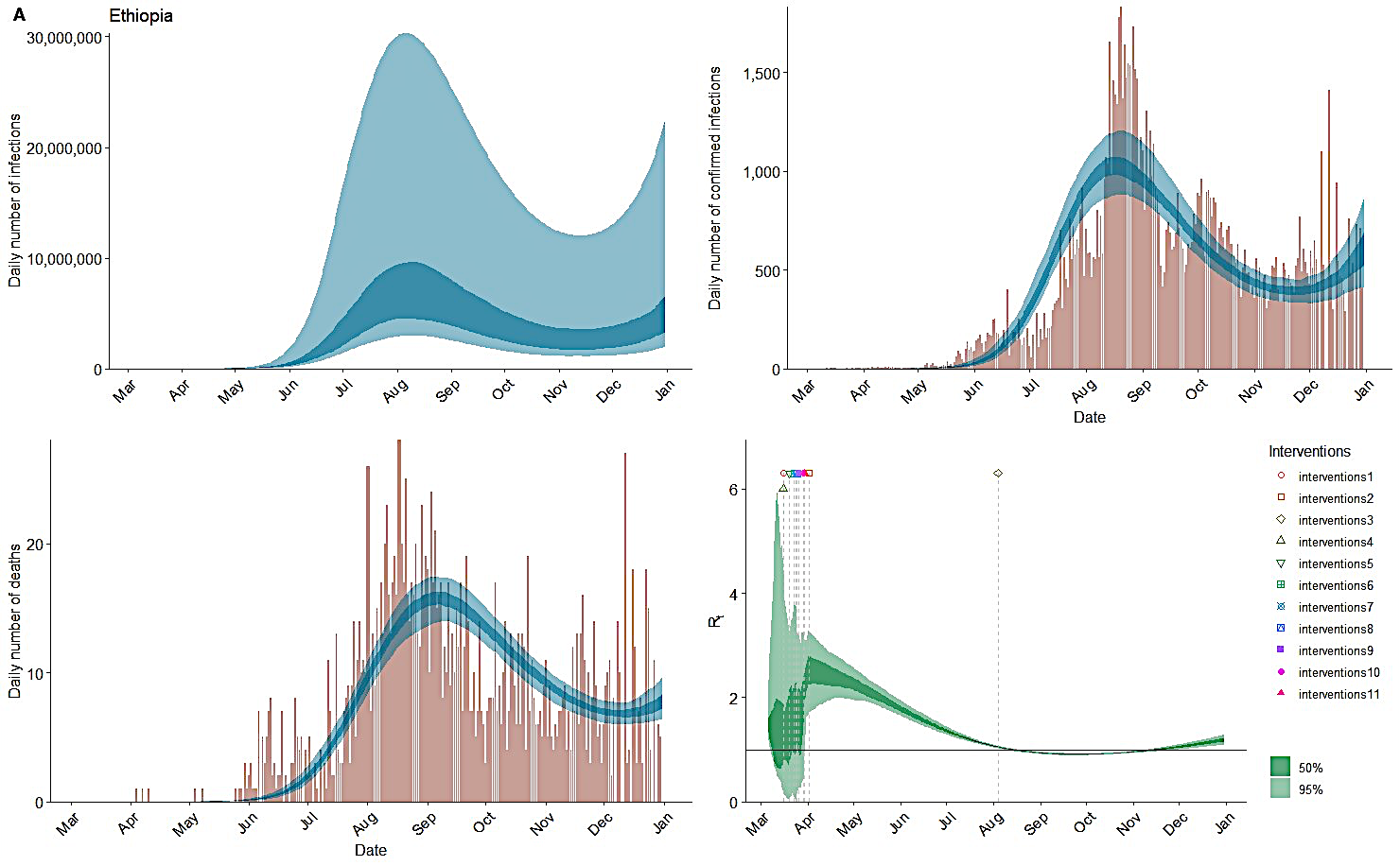


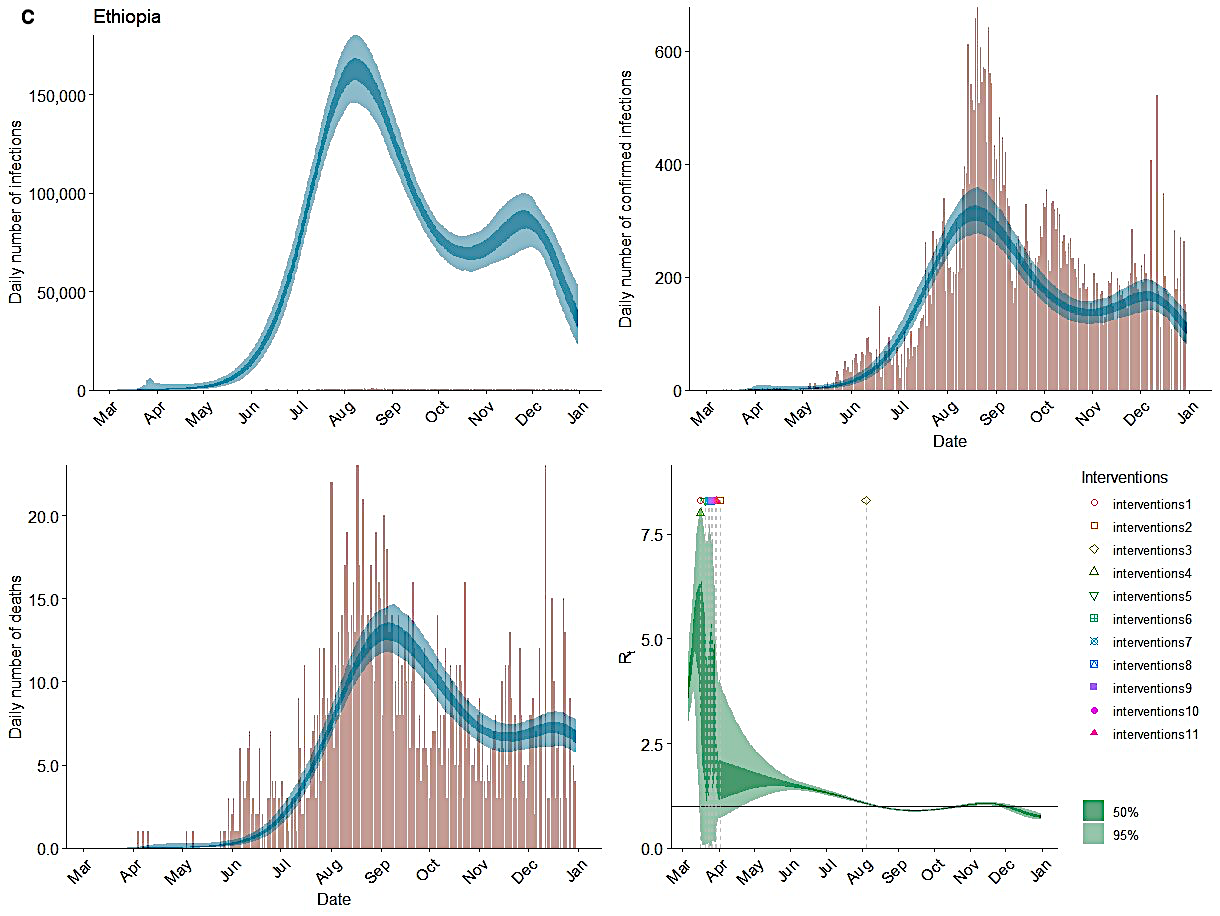


**Figure S2**. **Country-level estimates of infections, deaths and R_t_** **in Ethiopia**. *Scenarios*: A) The population is not BCG vaccinates, homogenous and not structured by age; B) BCG vaccinated population aged 39 years and below; C) BCG vaccinated population aged 40 years and above. *Top*: daily number of infections, brown bars are reported infections, blue bands are predicted infections, dark blue 50% credible interval (CI), light blue 95% CI. *Bottom-left*: daily number of deaths, brown bars are reported deaths, blue bands are predicted deaths. *Bottom-right*: time-varying reproduction number (R_𝒕_), dark-green 50% CI, light-green 95% CI.


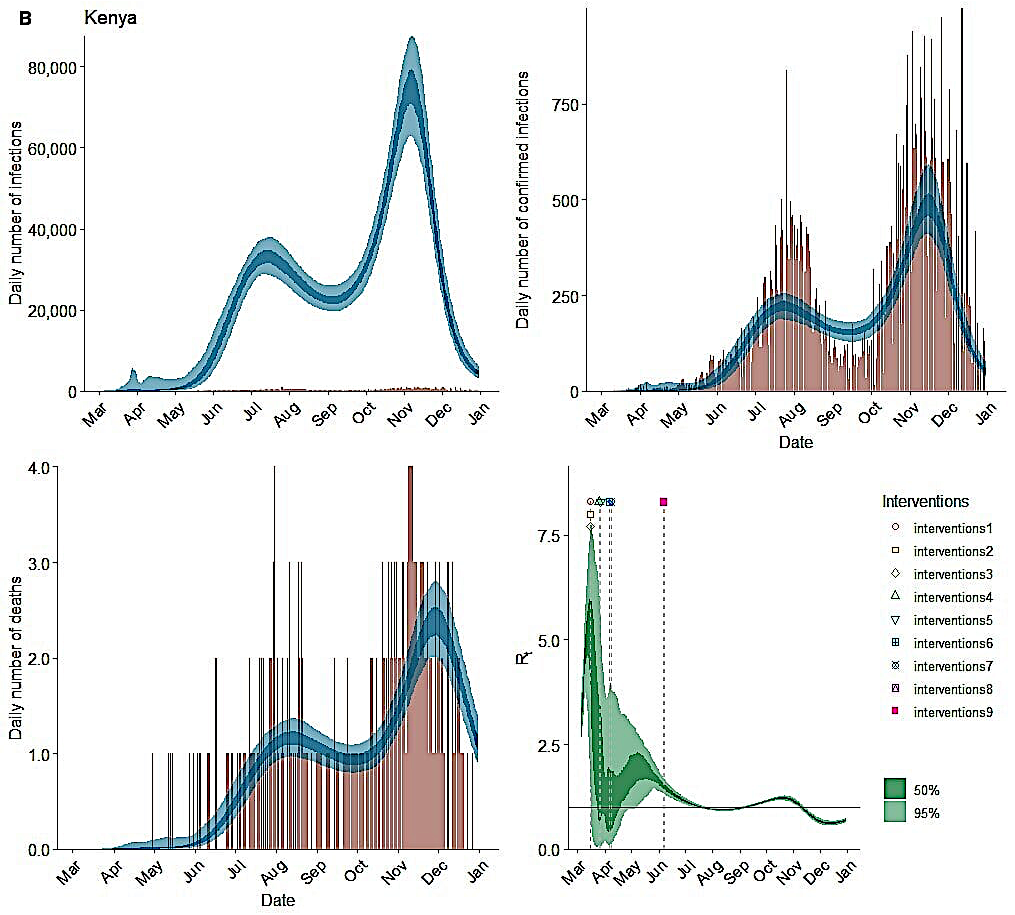


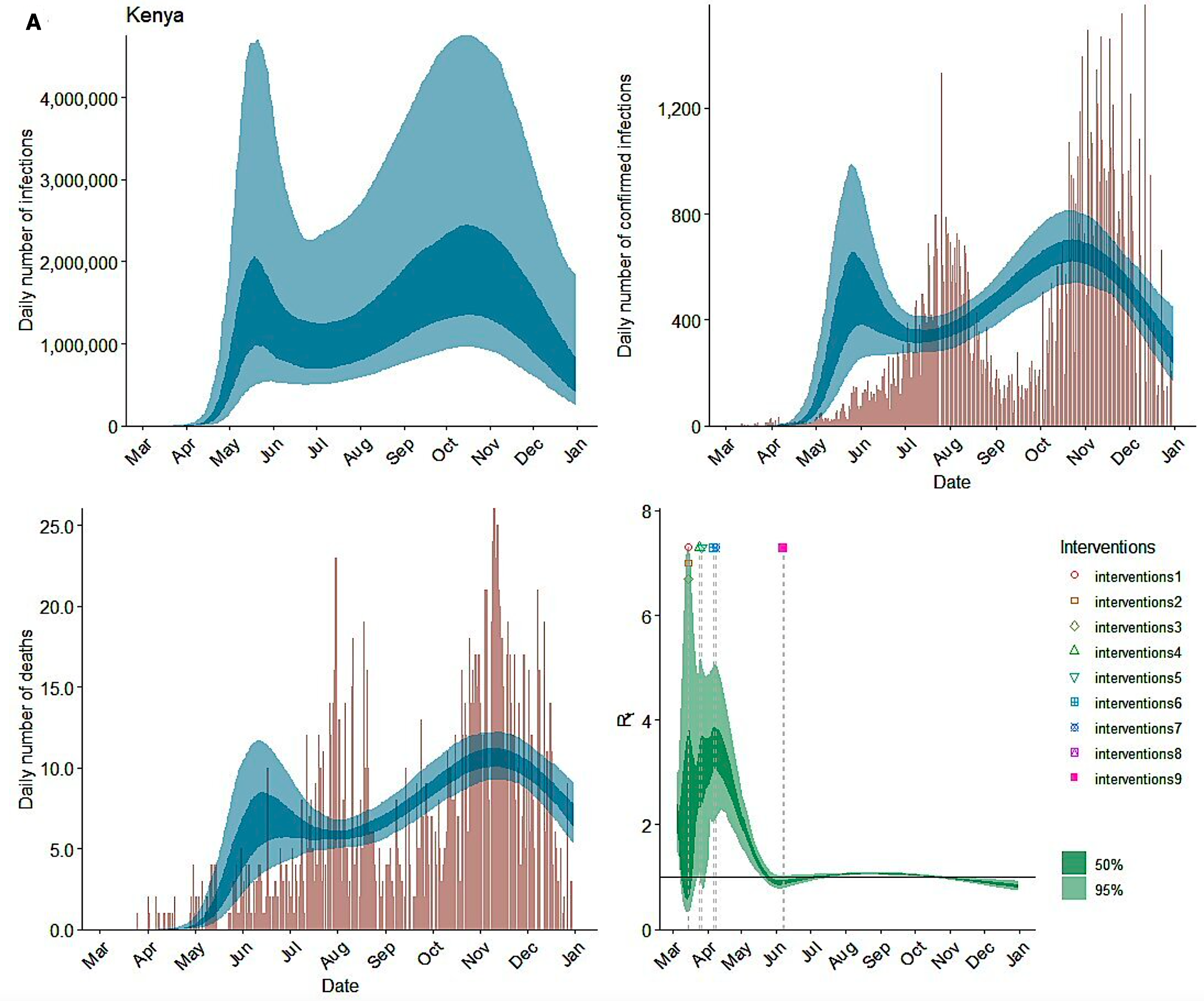


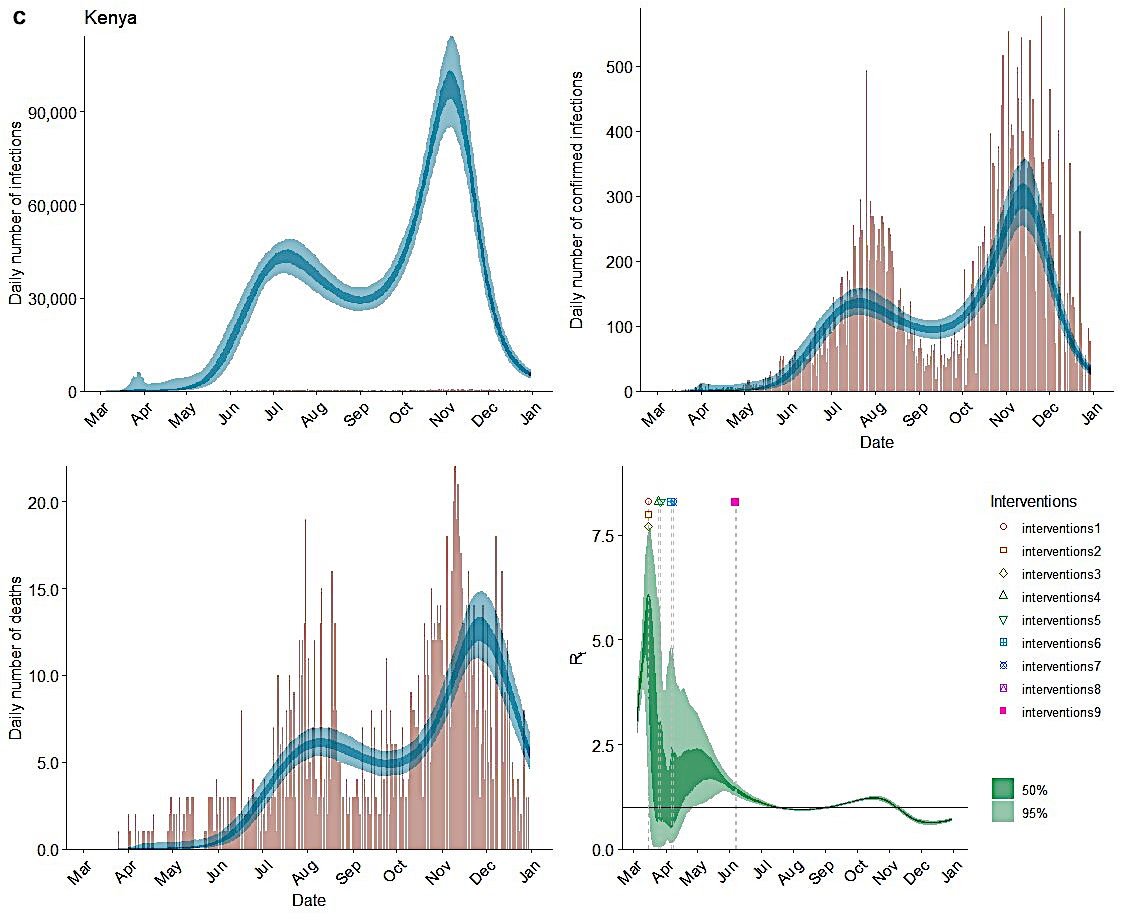


**Figure S3**. **Country-level estimates of infections, deaths and R_t_** **in Kenya**. *Scenarios*: A) The population is not BCG vaccinates, homogenous and not structured by age; B) BCG vaccinated population aged 39 years and below; C) BCG vaccinated population aged 40 years and above. *Top*: daily number of infections, brown bars are reported infections, blue bands are predicted infections, dark blue 50% credible interval (CI), light blue 95% CI. *Bottom-left*: daily number of deaths, brown bars are reported deaths, blue bands are predicted deaths. *Bottom-right*: time-varying reproduction number (R_𝒕_), dark-green 50% CI, light-green 95% CI.
